# Rearing conditions (isolated *versus *group rearing) affect rotenone-induced changes in the behavior of zebrafish (*Danio rerio*) embryos in the coiling assay

**DOI:** 10.1007/s11356-024-34870-x

**Published:** 2024-09-06

**Authors:** Rebecca von Hellfeld, Christoph Gade, Marcel Leist, Thomas Braunbeck

**Affiliations:** 1 Centre for Organismal Studies, Aquatic Ecology and Toxicology, University of Heidelberg, 69120 Heidelberg, Germany; 2 Present Address: School of Biological Sciences, University of Aberdeen, Aberdeen, AB24 3UU UK; 3 In Vitro Toxicology and Biomedicine, Department Inaugurated By the Doerenkamp-Zbinden Foundation, University of Konstanz, 78457 Constance, Germany; 4 CAAT Europe, University of Konstanz, 78457 Constance, Germany

**Keywords:** Locomotor assay, Rotenone, Developmental neurotoxicity, Spontaneous tail coiling, Rearing condition-dependence, Embryonic communication

## Abstract

Under regulations such as REACH, testing of novel and established compounds for their (neuro)toxic potential is a legal requirement in many countries. These are largely based on animal-, cost-, and time-intensive in vivo models, not in line with the 3 Rs’ principle of animal experimentation. Thus, the development of alternative test methods has also received increasing attention in neurotoxicology. Such methods focus either on physiological alterations in brain development and neuronal pathways or on behavioral changes. An example of a behavioral developmental neurotoxicity (DNT) assay is the zebrafish (*Danio rerio*) embryo coiling assay, which quantifies effects of compounds on the development of spontaneous movement of zebrafish embryos. While the importance of embryo-to-embryo contact prior to hatching in response to environmental contaminants or natural threats has been documented for many other clutch-laying fish species, little is known about the relevance of intra-clutch contacts for zebrafish. Here, the model neurotoxin rotenone was used to assess the effect of grouped versus separate rearing of the embryos on the expression of the coiling behavior. Some group-reared embryos reacted with hyperactivity to the exposure, to an extent that could not be recorded effectively with the utilized software. Separately reared embryos showed reduced activity, compared with group-reared individuals when assessing. However, even the control group embryos of the separately reared cohort showed reduced activity, compared with group-reared controls. Rotenone could thus be confirmed to induce neurotoxic effects in zebrafish embryos, yet modifying one parameter in an otherwise well-established neurotoxicity assay such as the coiling assay may lead to changes in behavior influenced by the proximity between individual embryos. This indicates a complex dependence of the outcome of behavior assays on a multitude of environmental parameters.

**Supplementary Information:**

The online version contains supplementary material available at 10.1007/s11356-024-34870-x.

## Introduction

Toxicological test methods providing detailed insight into modes-of-action and potential harmful effects by chemical compounds were first developed as a consequence of incidents such as the release of mercury into Minamata Bay (Yokoyama 2018), or the fetal survival and development effects of thalidomide (Vargesson 2015). Only recently, a stronger focus has been placed on potential neurological impacts, e.g., in the context of heavy metal or pesticide exposure (Andrade et al. 2017; Naughton and Terry 2018; Lee et al. 2019; Richardson et al. 2019; Vellingiri et al. 2022), now known as neurotoxicity. For developmental neurotoxicity (DNT) testing, there are methods to assess a chemical’s potential to impair the functioning of the developing nervous system, mainly by measuring immediate short-term responses (Selderslaghs et al. 2013).

Along with established and well-defined vertebrate organisms utilized in neurotoxicity (NT) studies, such as rats (*Rattus norvegicus*), rabbits (*Oryctolagus cuniculus*), or mice (*Mus musculus*) (Barrow and Clemann 2021), the zebrafish (*Danio rerio*) has become an emerging model organism with human health relevance (Kari et al. 2007; McGrath and Li 2008; Meyers 2018). The zebrafish is a low cost- and time-intensive model organism for laboratory research, which is suitable for various medium- to high-throughput assays, as well as offering biological benefits such as the thoroughly described ex utero development in a transparent chorion with well-defined stages (Kimmel et al. 1995; Brustein et al. 2003; Selderslaghs et al. 2010; De Esch et al. 2012; Ramlan et al. 2017). Most importantly, due to the delayed onset of exogenous feeding, early developmental stages of the zebrafish are not regarded as protected under current EU animal testing regulations (EC 2010) and thus comply with the 3Rs principle (reduce, replace, refine) (EC 2010)This means that—from a legal point of view—experiments with zebrafish do not require permission for the first 5 days of its development (Strähle et al. 2012), and are considered compliant with the 3Rs principle (Russell and Burch 1959). Thus, as an ‘alternative in vivo system’, the zebrafish embryo allows for a direct link of the changes observed to the developmental stage of the individual, allowing a better understanding of the effects of a chemical (Schier et al. 1996; Hjorth and Key 2002; Kalueff et al. 2013).

The embryonic development of the zebrafish is largely similar to that of mammals in terms of, e.g., morphology, along with similarities in their genome and compound uptake and metabolism (Howe et al. 2013; Pelka et al. 2017). For example, in mammals, the neurotransmitter acetylcholine (ACh) is vital for the differentiation of neural cells and is found in the developing neural plate (Sam and Bordini 2022). In the developing zebrafish, ACh is detected from approx. 17 h post-fertilization (hpf), at which time initial involuntary muscle contractions in the tail can be observed (Grunwald et al. 1988; Melançon et al. 1997). These are attributed to the presence of ACh as well as a limited electrically coupled spinal cord network (Saint-Amant and Drapeau 1998; Brennan et al. 2005). At around 20 hpf, this is followed by the expression and activation of glycine receptors, as well as glutamatergic and γ-aminobutyric acid (GABA)-ergic neurotransmitters (Saint-Amant and Drapeau 1998; Moly et al. 2014; Wirbisky et al. 2014). This coincides with the onset of the so-called touch response in the developing zebrafish embryo (from around 21 hpf onwards; Brustein et al. 2003), which has been attributed to the emergence of chemical synapse functionality (Pietri et al. 2009).

Considering the interdependence of these developmental steps and the sensitivity of the nervous system to perturbation, exposure to chemicals might thus influence the timeline and/or development of various neurotransmitters in the zebrafish embryo, resulting in persistent effects on neurodevelopment and behavior. To this end, novel behavioral assays aimed to quantify early locomotive behavior utilized the well-defined neuro-developmental timeline to establish a connection between external stimuli (such as light, chemical exposure, or mechanical stimuli) and observed physical response (such as coiling and swimming behavior, or social responses; see e.g., Selderslaghs et al. 2010; Colwill and Creton 2011; Basnet et al. 2019). These approaches are based on group-reared embryos. In refining experimental approaches, abiotic factors such as temperature, light, and chemical exposure regime have been shown to have profound impact on early development of zebrafish (Schirone and Gross 1968; Villamizar et al. 2013). While effects of environmental stressors on embryos have been studied thoroughly, it has been suggested that reciprocal embryo-to-embryo stimulation might have just as much of an impact on early development (Noguera and Velando 2019). This pre-hatching communication has mostly been associated with mechanical stimuli through direct contact, a phenomenon which has been observed in various (aquatic) organisms (Nishide and Tanaka 2016). For example, embryos of the viperine water snake (*Natrix maura*) communicate with faint vibrations caused by their own heartbeat as a cue for their metabolic level, allowing embryos of different stages to align and synchronize development and hatching (Aubret et al. 2016), which is also known as the catch-up hypothesis (Doody 2011). Such insights underline the need to study effects on clutches, since embryos are also thought to obtain information from one another about the quality of the natal environment (Aubret et al. 2016; Noguera and Velando 2019).

In a recently published study, the coiling assay as developed by Zindler et al. (2019) was used to highlight the suitability of the assay to neurotoxicants beyond those affecting the ACh pathway, as well as the assay’s potential to identify compound-specific behavioral patterns (von Hellfeld et al. 2023). The present study aims to improve the current understanding of the potential impact of rearing conditions on the development of behavioral patterns in zebrafish embryos. To this end, the coiling assay was conducted as previously described in Zindler et al. (2019) and modified to inhibit direct contact between individual embryos. The mitochondrial complex 1 inhibitor rotenone, a compound frequently used to induce Parkinson’s disease for research purposes (le Couteur et al. 1999; Betarbet et al. 2000), was used as a known neurotoxicant leading to behavioral changes. The effects of isolated versus batch rearing conditions on the mean duration of tail coiling and initiation between 21 and 47 hpf were recorded, utilizing the change of light conditions at 23.5 and 37.5 hpf as an external stimulus.

## Material and methods

### Chemicals and test concentrations

Rotenone was obtained from TCI (Eschborn, Germany) and distributed by the Joint Research Centre in Ispra with shipping and storage in accordance with the manufacturer’s instructions. Dimethyl sulfoxide (DMSO) was obtained from Honeywell (Offenbach, Germany). Stock solutions were prepared in DMSO and stored at 4 °C during the experiment. The final test solutions (0, 1, 10.1, 20.2 nM) were prepared freshly prior to each experiment, using standardized water as specified by the Organization for Economic Co-operation and Development test guideline (OECD TG) 236 (OECD 2013) with a final DMSO concentration of 0.1%; test solutions were renewed daily.

### Fish maintenance and handling

Adult wild-type Westaquarium strain zebrafish (*Danio rerio*) from the Aquatic Ecology and Toxicology Group at the University of Heidelberg, Germany (license number: 35–9185.64/BH), were used for breeding. Published protocols for fish maintenance and egg collection were followed, beginning with the collection 30 min after light onset (Lammer et al. 2009). The quality of eggs was assessed prior to the selection of embryos for exposure, no later than 1.5 h after spawning (Kimmel et al. 1995; OECD 2013).

### The fish embryo toxicity (FET) test (OECD TG 236)

The acute toxicity of rotenone was determined using the protocol of OECD TG 236 (OECD 2013), in accordance with a previously published study (von Hellfeld et al. 2020). Here, fertilized eggs were assessed for viability and transferred into pre-exposure dishes containing 5–81 nM rotenone, the negative control (artificial water; OECD 2013), the solvent control (0.1% DMSO), or the positive control (25 mM 3,4-dichloroaniline; DCA) before being transferred to 24-well plates. Per well plate (i.e., independent replicate), one embryo was placed in each well (*n* = 24, of with *n* = 4 internal negative control and *n* = 20 for medium or controls). Prior to medium change, embryos were examined daily. A full description of the methods and results can be found in von Hellfeld et al. (2020).

### The coiling assay

**Fig. 1:**
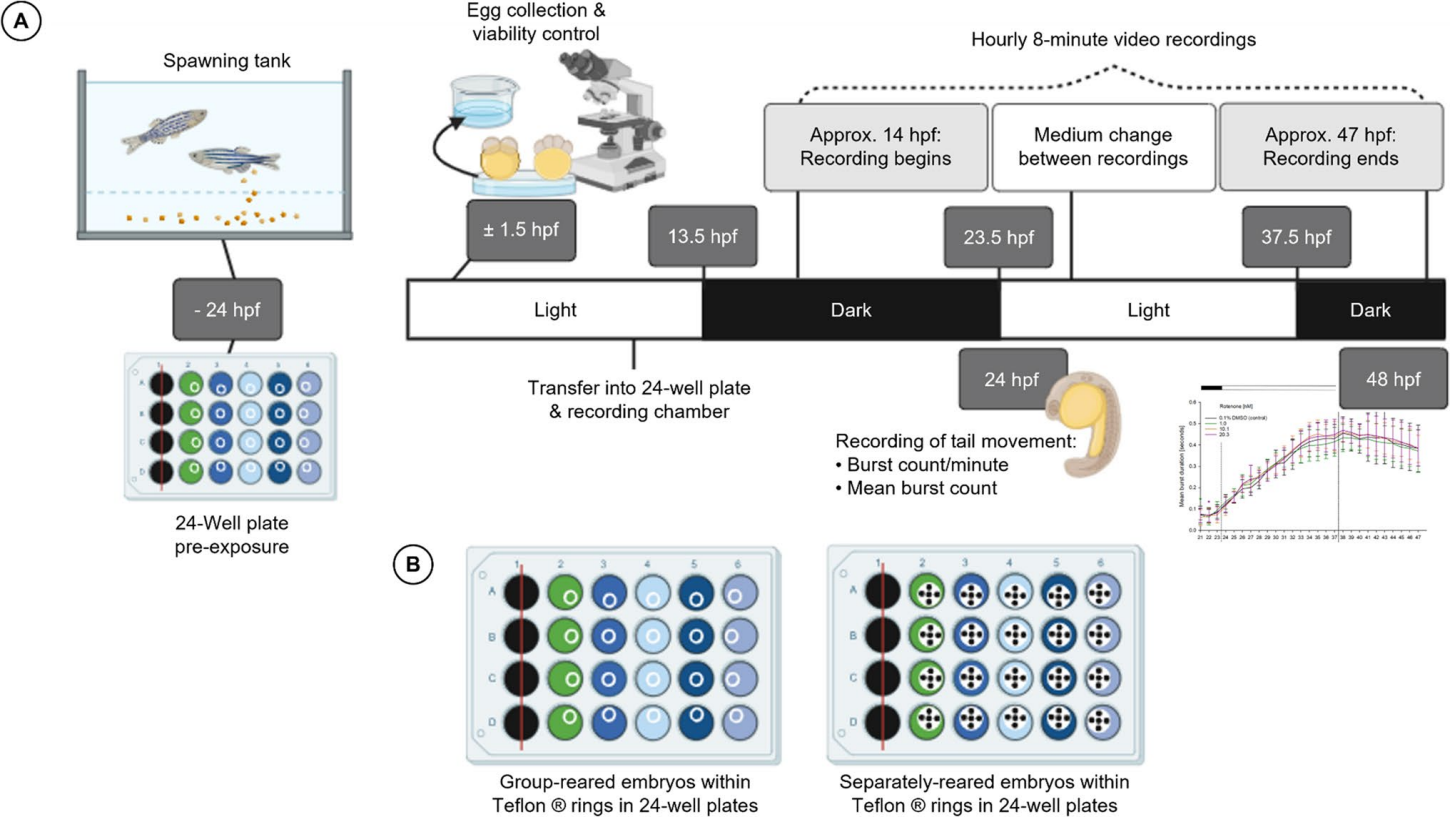
Timeline of the zebrafish (*D. rerio*) embryo coiling assay and setup of well plates. **A** Spawning units are set up 24 h pre-exposure, and the 24-well plates are pre-exposed to the test solutions for 24 h. Fertilized eggs are collected the following morning and transferred to the 24-well plates. The medium is renewed completely every 24 h. Endpoints assessed are burst counts per minute and mean burst duration (sec). **B** The two alternative layouts of the wells within the 24-well plates: group rearing (left): Teflon® rings for group rearing of 5 zebrafish embryos; separate rearing (right): Teflon® plates with 5 inlets (•) for separate rearing of individual zebrafish embryos

The embryos were treated according to Zindler et al. (2019), where fertilized embryos were transferred into the rotenone test solutions (1.0, 10.1, and 20.3 nM) or the solvent control (0.1% DMSO) for further development at 26.0 ± 1.0 °C. At 8 hpf, embryos were transferred to pre-exposed 24-well plates (TPP, Trasadingen, Switzerland). For group-reared exposure, three independent replicates were conducted with polytetrafluoroethylene rings (PTFE, ESSKA, Hamburg, Germany) herein referred to as Teflon® rings of approximately 0.2 mm in height and 5.3 mm in diameter, to secure the embryos within the ring (total of 60 embryos per exposure concentration). Here, only 5 of the 6 columns of each well plate (i.e., independent replicate) could be used, due to the camera frame (see Fig. 1 for clarification). Each column was asigned an exposure concentration (or solvent control) at random, and each well-contained *n* = 5 embryos, thus resulting in *n* = 20 embryos per concentration per independent replicate. For exposure of separately reared animals, three replicates were conducted as described above, replacing the Teflon® ring by a 5.3-mm-diameter Teflon® plate equipped with 5 inlets, each with a diameter of approx. 1 mm (precision mechanics workshop, Center for Organismal Studies, University of Heidelberg). The Teflon® rings and plates had been pre-cleaned and stored in ethanol prior to use (Fig. 1).

The 24-well plate was placed on an acrylic glass table with infrared lights (880 nm, Knightbright-Europe; Issum, Germany) in an incubator at 26.0 ± 1.0 °C set to a light/dark cycle of 14/10 h. To avoid mechanical disruption, the incubator was automated to switch off 3 min prior to the onset of recording. Zebrafish embryo behavior was recorded for 8 min/h hourly between 21 and 47 hpf (MPEG-4, 25 frames/s) with an acA1920-155 µm camera equipped with an M7528-MP F2.8 f75 mm lens (Basler, Ahrensburg, Germany) and an RG 850 filter (heliopan, Gräfelfing, Germany) using Ethovision™ XT 11.5, (Noldus, Wageningen, Netherlands).

The test solutions were renewed daily, returning the 24-well plate into the incubator at least 20 min before the next recording to allow the embryos to re-acclimatize. The videos were analyzed with DanioScope™ 1.1 (Noldus, Wageningen, Netherlands), assessing the mean burst count per minute and the mean burst duration (sec). Individual embryos expressing excessive (i.e., non-recordable) hyperactivity were excluded from assessment at the given time point (see Fig. SM 1 and discussion of the phenomenon in von Hellfeld et al. (2023)) to facilitate the data acquisition from DanioScope™. The program parameters were 2% “activity onset,” 0.5% “activity offset,” 100 ms “minimum inter peak interval,” and 0 ms “minimum peak duration.”

### Statistical analysis

Lethal (LC) and effect (EC) concentrations were calculated for OECD TG 236 data, with computing effect levels of 10 and 50% based on probit analysis using linear maximum likelihood regression with ToxRat® 2.10.03 (ToxRat™ Solutions, Alsdorf, Germany); both lethal and sublethal effects were included into the calculation of EC values (Braunbeck et al. 2015). All statistical analyses for the coiling data were conducted with GraphPad Prism™ 6 (Dotmatics, USA). One-way analysis of variance (ANOVA) on ranks (Kruskal–Wallis test) analyzed the differences in coiling frequency between treatment groups and negative controls, followed by Dunn’s post hoc test against negative controls. Analyses were carried out separately for each replicate and considered to distinguish between biological variability and actual exposure-related behavioral effects (Zindler et al. 2019). Statistically significant alterations (*p* < 0.05) in at least two replicates were considered an exposure-related effect rather than biological variability. Additionally, the behavioral change from 37 to 38 hpf for all treatment groups was analyzed in the same manner, but utilizing the Wilcoxon matched-pairs signed rank test. To examine the difference between behaviors of separately and group-reared individuals of the control groups, the raw behavioral values were assessed in a two-tailed Mann–Whitney *U* test. Graphs were created with SigmaPlot™ 13.0.0.83 (Systat Software, Inpixon, Chicago, USA); the layout was edited with Inkscape 1.0.1 (https://inkscape.org).

## Results and discussion

### Acute and sublethal toxicity of rotenone in the FET test with zebrafish embryos

The FET test results from our previous work with rotenone determined the EC and LC values for rotenone in the zebrafish embryo to be as follows: 9.3 ± 0.4 nM EC_10_, 18.3 ± 0.8 nM EC_50_, 15.6 ± 4.4 nM LC_10_, and 30.0 ± 6.8 nM LC_50_ (von Hellfeld et al. 2020). The following endpoints were recorded during the previously conducted FET tests: coagulation; lack of tail detachment and delayed development from 24 hpf; reduced or missing heartbeat, edema, increased movement, and reduced pigmentation from 48 hpf; reduced hatching at 96 hpf (von Hellfeld et al. 2020). The EC and LC values presented here were in line with those published by Melo et al. (2015) as well as data obtained from other standardized fish tests (Marking and Bills 1976). However, Kalyn et al. (2019) determined significantly lower toxicity in their FET test, with a 96 hpf LC_50_ of 50 nM. Although the FET test only determines the teratogenic potential of a chemical, endpoints such as coiling or external stimuli response in conjunction with an understanding of the underlying mode-of-action may be interpreted as a sign of neurotoxicity (von Hellfeld et al. 2020, 2023). In the case of rotenone, this hypothesis is supported by Byrnes et al. (2018), who concluded that embryonic exposure to rotenone induced brain malformations and yolk extension delays in zebrafish embryos. Others have linked behavioral changes from rotenone exposure to decreased brain dopamine levels (Wang et al. 2017). A detailed discussion of the teratogenic effects of rotenone can be found in von Hellfeld et al. (2020).

### Effects of rotenone are more severe in group-reared zebrafish than in separately reared embryos

Based on the severe toxic effects on the behavior of zebrafish embryos observed after rotenone exposure published by von Hellfeld et al. (2023), the present study was initiated to elucidate potential impact of rearing conditions on effects by rotenone. Due to excessive motion associated with frequent trajectory crossing of tracks which could not reliably be resolved during analysis, individual zebrafish embryos had to be excluded from ≥ 30 hpf (see SI 1 and SI 2).

**Fig. 2:**
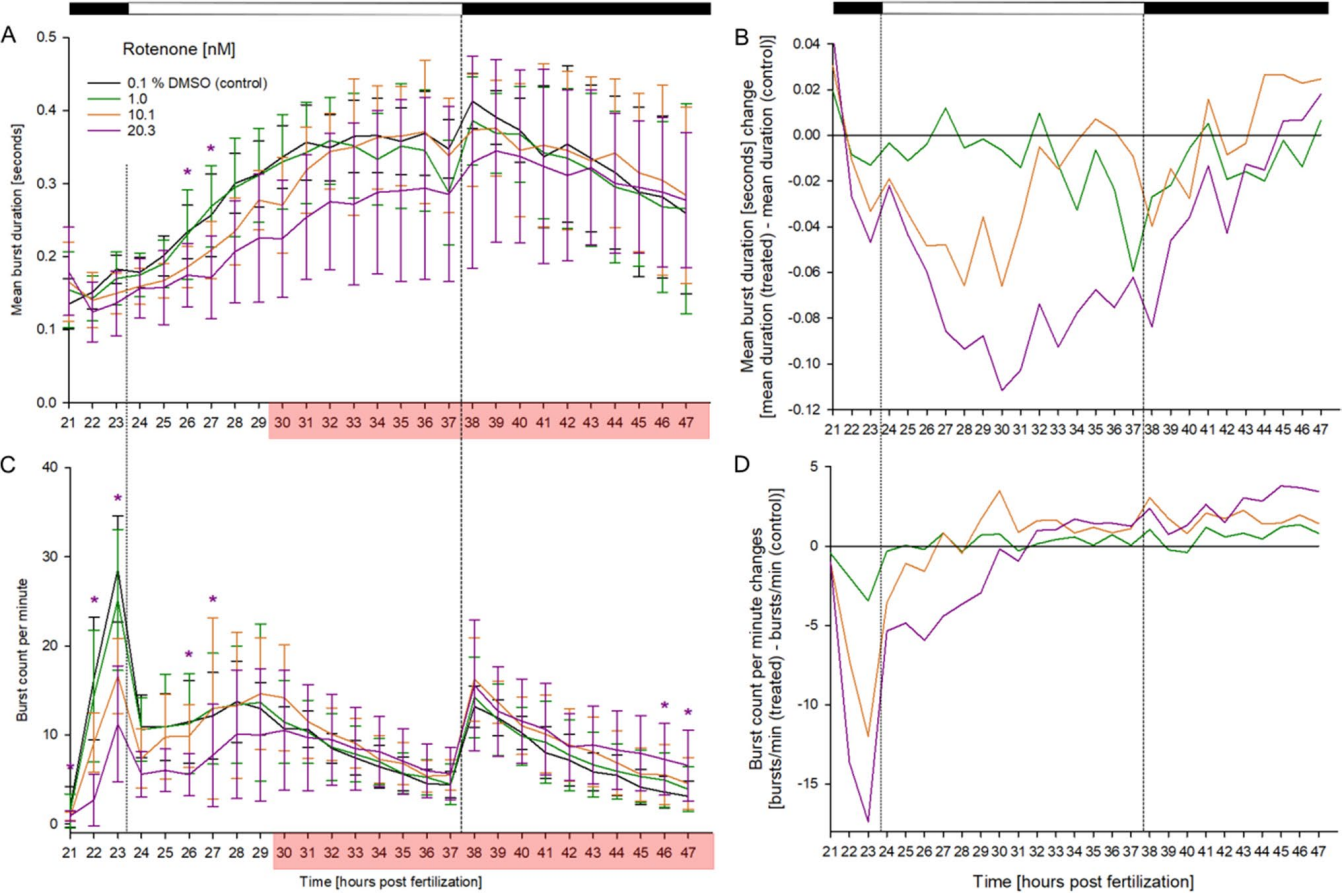
Effect of rotenone on spontaneous tail movement of group-reared zebrafish (*D. rerio*) embryos during the light/dark cycles of the coiling assay: The mean burst duration [seconds] (**A**) and normalized burst duration (**B**), as well as the burst count per minute (**C**) and normalized burst count per minute (**D**) between 21 and 47 hpf is shown (*n* = 3, 20 embryos per concentration/replicate). **A**, **C** Data are given as means ± standard deviation; **B**, **D** normalized data adjusted to negative control group. Top bar: Light cycle phases (black = dark; white = light); *Time point and concentration (in corresponding color) of significant difference to controls (for *p*-values, see Table SI 3). Red box: ≥ 20% of individuals were excluded from analysis in at least one exposure concentration at this time point. Note: This figure was previously published by von Hellfeld et al. (2023) with permission of the publisher (article published under CCA 4.0 international license) and consent of all authors. It was included in the present publication to highlight differences with separately reared embryos shown in Fig. 3

Group-reared embryos exposed to rotenone that could successfully be tracked showed decreases in both mean burst duration (Fig. 2A, B) and burst count per minute (Fig. 2C, D), which were statistically significant in individuals exposed to 20.3 nM pre-30 hpf. While no other coiling assay data could be found in peer-reviewed literature, previous research had shown rotenone exposure of 6-day-old zebrafish, as well as adult fish, to cause a similar decrease in total distance and duration moved in swimming assays (Wang et al. 2017; Kalyn et al. 2019; Hettiarachchi et al. 2022). However, there is some uncertainty about rotenone effects in adult zebrafish since Wang et al. (2017) reported an increase in swimming duration under light conditions.

**Fig. 3:**
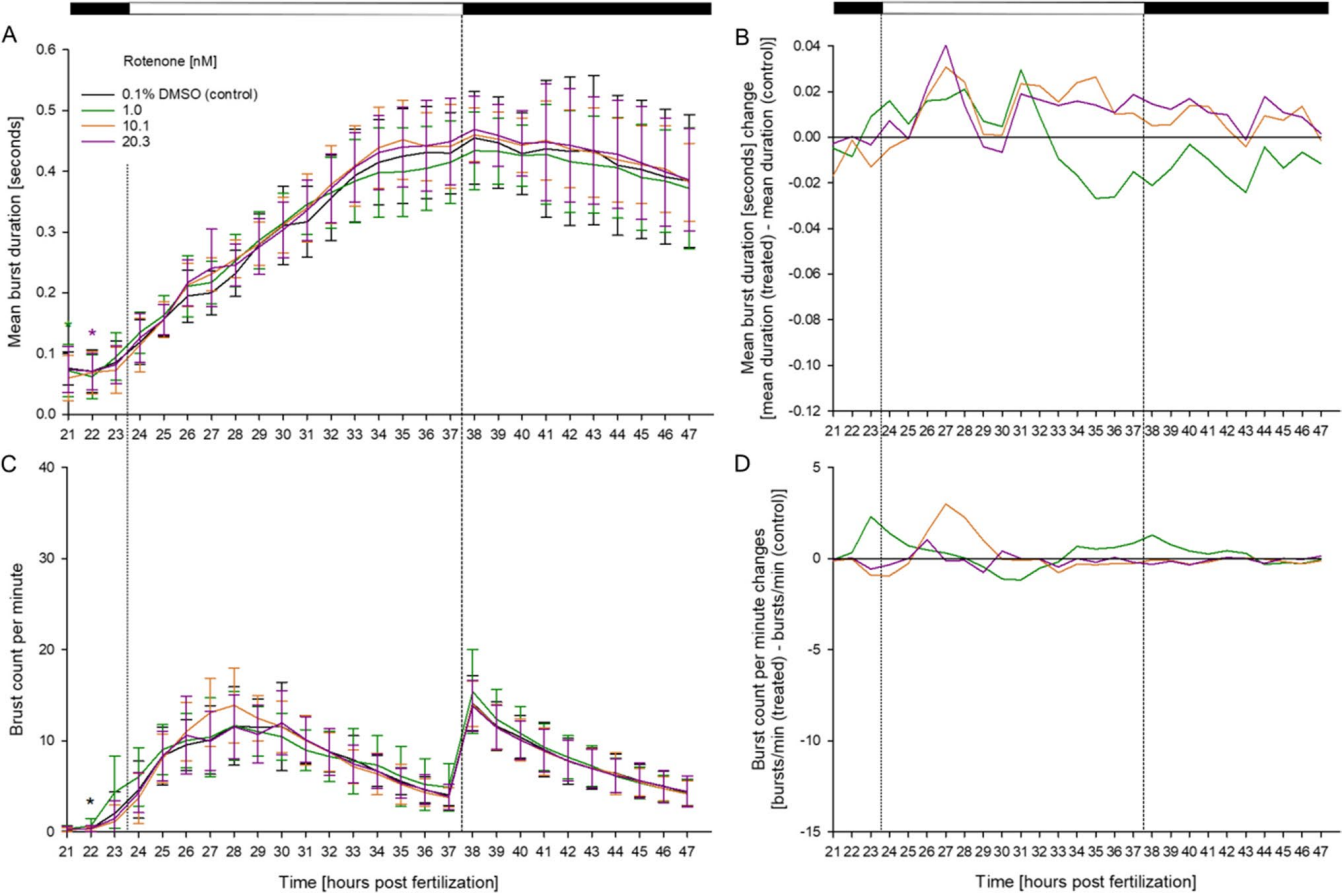
Effect of rotenone on spontaneous tail movement of separately reared zebrafish (*D. rerio*) embryos during the light/dark cycles of the coiling assay. The mean burst duration [seconds] (**A**) and normalized burst duration (**B**), as well as the burst count per minute (**C**) and normalized burst count per minute (**D**) between 21 and 47 hpf is shown (*n* = 3, 20 embryos per concentration/replicate). **A**, **C** Data are given as means ± standard deviation; **B**, **D** normalized data adjusted to control group; Top bar: Light cycle phases (black = dark; white = light); *Time point and concentration (in corresponding color) of significant difference to controls (for *p*-values, see Table SI 3)

The excessive movement observed in rotenone-exposed zebrafish embryos led to a form of movement, which was conspicuously different to control individuals. Since DanioScope™ was unable to distinguish between embryos with overlapping trajectories (thus leading to potentially erroneous data), Teflon® plates with 1-mm-diameter inlets for a total of 5 embryos were utilized instead of the Teflon® rings. These plates eliminated the need to remove individual zebrafish embryos from observations by not allowing their trajectories of movement to coincide. However, in these separately reared embryos, no statistically significant deviations in burst duration or burst count per minute compared to the control group were observed (A, C). Data normalized to controls indicate that all treatment groups showed similar burst durations and burst counts per minute as the control group, with exposure to 1.0 nM slightly suppressing the burst duration (B, D). Only the initial peak in the burst count per minute at around 23 hpf, observed in group-reared individuals (C), was missing in the exposure-negative control individuals of the separately reared embryos (see the “Rearing conditions also affect behavior in unexposed zebrafish embryos” section for details)*.*

**Fig. 4:**
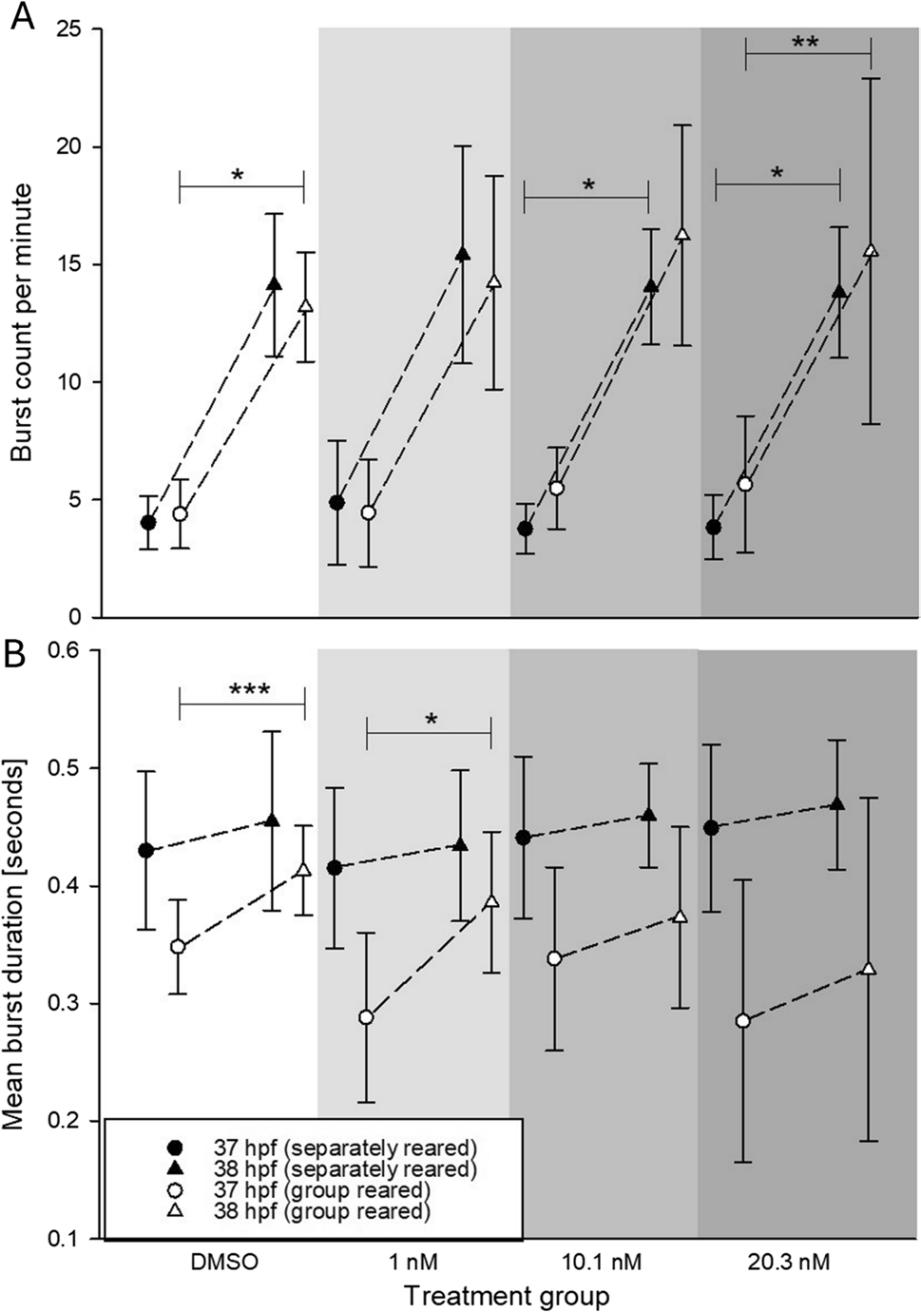
Effect of rotenone exposure on mean burst duration [seconds] (**A**) and burst counts per minute (**B**) in the coiling assay with group- (non-filled symbols) and separately reared (filled symbols) zebrafish (*D. rerio*) embryos during the illumination change at 37.5 hpf (circles: 37 hpf, triangles: 38 hpf). The grey scale indicates the different treatment groups for visualization purposes. *N* = 3, 20 embryos per concentration/replicate. Statistically significant difference in the coiling behavior between the two time points within one treatment group and rearing condition, based on the Wilcoxon matched-pairs signed rank test (* < 0.1, ** < 0.05, *** < 0.005). A table of *p*-values can be found in Table SI 4

The analysis of coiling before and after the second dark phase (37.5 hpf) by rotenone-exposed individuals revealed a seeming induction of a significant change in behavior under both rearing conditions. In group-reared individuals, exposure to DMSO and 1 nM rotenone-induced a statistically significant increase in mean burst duration after the onset of the second dark phase, whereas groups exposed to higher concentrations did not show such an increase in activity. However, at this time point, many of the group-reared embryos were already excluded from analysis due to the previously highlighted non-trackable excessive movements. In contrast, no significant change in the mean burst duration upon the second dark phase was observed in separately reared zebrafish embryos (Fig. 4A). The burst count per minute in group-reared embryos was statistically significantly increased in the control group and individuals exposed to 20.3 nM rotenone. Separately reared individuals exposed to ≥ 10.1 nM rotenone also expressed significantly elevated burst counts per minute after the onset of darkness (Fig. 4B).

### Rearing conditions also affect behavior in unexposed zebrafish embryos

**Fig. 5:**
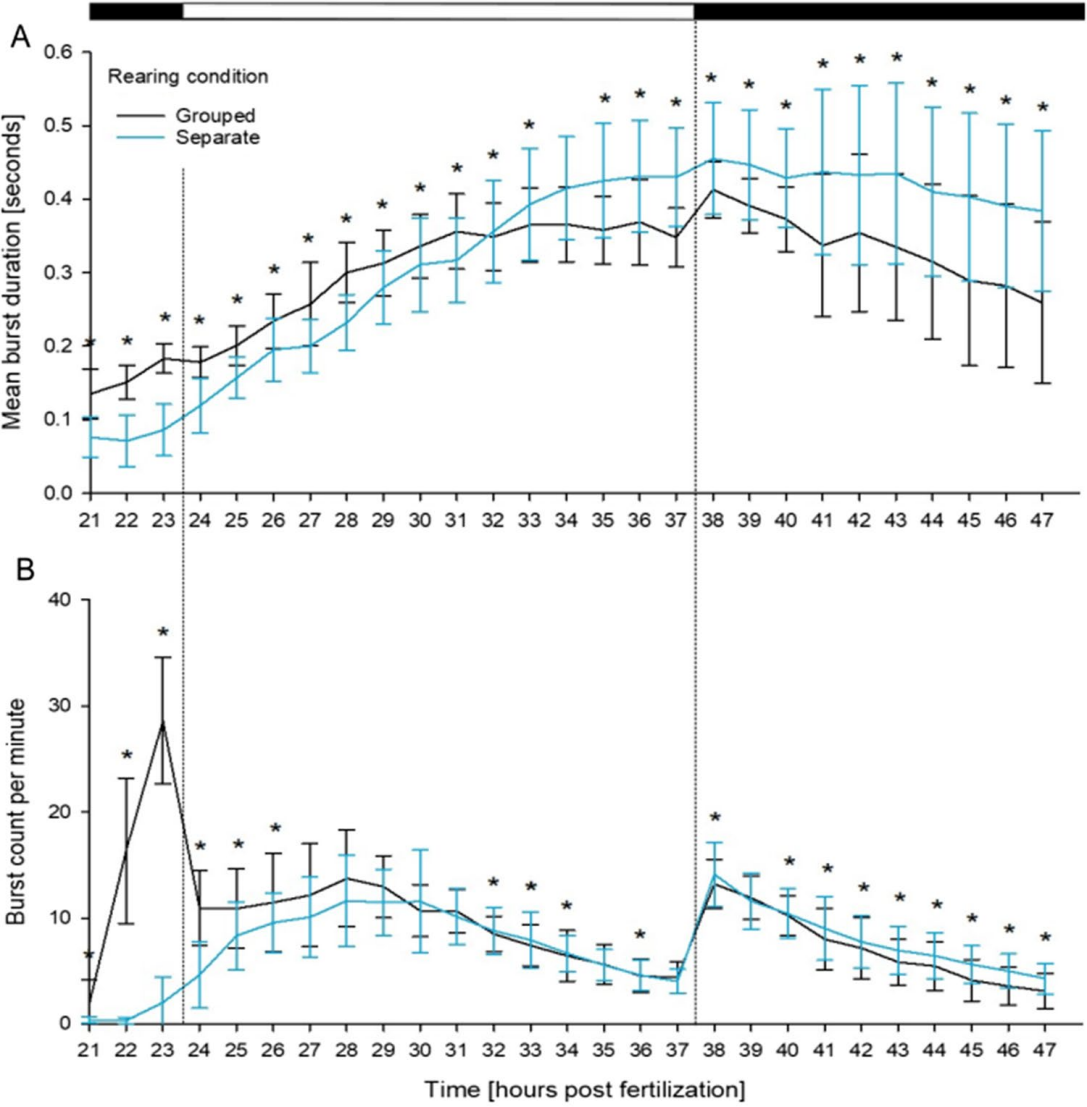
Effect of rearing conditions on mean burst duration [seconds] (**A**) and burst count per minute (**B**) in group- (black) and separately reared (blue) zebrafish (*D. rerio*) embryos (control group, 0.1% DMSO) in the coiling assay between 21 and 47 hpf (*n* = 3, 20 embryos per replicate). Data are given as means ± standard deviation; Top bar: Light cycle phases (black = dark; white = light); *Time point of significant difference between the two groups, based on the Mann–Whitney *U* test. A table of all significant *p*-values can be found in Table SI 5

Overall, both rearing conditions led to a similar pattern of mean burst duration characterized by a transient increase in mean burst duration until approx. 38 hpf followed by a reduction in later developmental stages. However, mean burst duration in separately reared embryos was less than half as frequent as those in group-reared embryos (exception: same frequencies at 32 hpf). After 32 hpf, separately reared embryos clearly showed longer burst durations (Fig. 5A). A similar albeit more obvious trend could be observed in the burst count per minute, where separately reared zebrafish embryos initially proved significantly less active, before reaching and exceeding the burst count per minute expressed by group-reared individuals. Moreover, during these initial periods of coiling, the normal initial peak in activity at ~ 24 hpf was absent in separately reared zebrafish embryos (Fig. 5B).

It is known that the formation of GABA-dependent synapses precedes that of glutamatergic ones and that GABA-dependent synapses are initially excitatory, generating a basic activity pattern that aids the neuronal growth and formation of synapses (Ben-Ari 2002). In later development, glutamatergic neurotransmitters are thought to drive the touch response in zebrafish embryos (Saint-Amant and Drapeau 2001). Drapeau et al. (2002) hypothesized that the initial activity peak (here seen at 23 hpf in the burst count per minute) may be an indicator of active glycinergic and glutamatergic neurotransmitter integration into coiling behavior, and that movements observed prior to this are a response of primal scaffolding neurons, rather than an organized response to a stimulus (Saint-Amant and Drapeau 1998).

The excitatory glutamatergic neurotransmitter is also known to be required for the double coiling pattern that can normally be observed from 26 hpf and is considered important for successful hatching and effective swimming (Roussel et al. 2021). The lack of an initial coiling activity peak in the separately reared embryos in the present study without any macroscopically visible developmental alterations or reduced hatching rates (details not shown in von Hellfeld et al. 2020) could be attributed to the lack of contact between embryos leading to a lack of stimulation of the glutamatergic neurotransmitter pathway. It may also be hypothesized that the development of this pathway is only delayed, with the pre-natal behavioral changes reported here remaining without consequences for subsequent hatching and survival in later developmental stages.

### How does rotenone exposure affect zebrafish embryo behavior?

Considering the present data, it should be considered that neurotoxic effects by rotenone could not be documented for separately reared embryos exposed to the same concentration and under otherwise unchanged conditions. Previous research also revealed that group-reared zebrafish embryos were more active after the first 5 days of development than separately reared embryos (Zellner et al. 2011). Therefore, Zellner et al. (2011) proposed that rearing conditions might also influence the effects of neuroactive substances (i.e., compounds that induce a reversible behavioral change due to exposure; Ogungbemi et al. 2019). This hypothesis found support by previous research showing that group rearing, through its increased stimulation, can facilitate connectivity between neurons (Lazic et al. 2006). This may provide an insight into the overall lack of behavioral observations in embryos reared separately in the present study. As conditions of the presented experimental setup were otherwise unchanged between the rearing scenarios, differences in e.g., oxygen saturation were excluded as causes for the behavioral deviations. This further consolidates the hypothesis that pre-hatching communication between zebrafish embryos plays a vital role in early behavioral expression and can, in fact, be measured in the coiling assay.

## The wider context: unhatched embryo-to-embryo communication: visual, auditory, or mechanical?

The observations presented suggest a potential involvement of some form of communication between individual embryos during development. Three pathways may possibly be involved: the visual registration of movement, changes detected via the auditory system, or sensing movement in the water via the lateral line.

Although zebrafish eyes begin to develop at ~ 10 hpf, the only differentiate into retinal ganglion cells and the optic nerve by ~ 28 hpf and are fully formed by 72 hpf, visual registration of small-scale changes such as individual movements are likely not involved in embryo communication (Glass and Dahm 2004; Morris and Fadool 2005). Optokinetic responses in zebrafish can first be measured around the time they begin to hatch and become as precise as the response measured in adults by 96 hpf (Easter and Nicola 1997). It has been postulated that a visual startle response can be detected at ~ 68 hpf, and the light–dark response becomes evident not much later (Easter and Nicola 1996; Morris and Fadool 2005). However, as the coiling assay assesses embryos between 21 and 47 hpf, eyes—although present—does not seem to be developed enough to be involved in stimulus perception.

In zebrafish, hearing requires the existence of the inner ear (consisting of multiple macular compartments within the otolith), of the swim bladder, of Weberian ossicles, and of perilymphatic spaces (Popper and Fay 1973; De Esch et al. 2012). Although the inner ear begins to develop at 16 hpf, the swim bladder does not develop fully until approximately 96 hpf (Hernandez et al. 2018). Moreover, while 7-day-old zebrafish larvae are capable of perceiving and responding to audiograms (Haddon and Lewis 1996), younger zebrafish only expressed escaping behavior but no hearing abilities (Zeddies and Fay 2005; Lu and Desmidt 2013). It may thus be assumed that stimuli perceived in early developmental stages are not transmitted through the auditory system.

In zebrafish embryos, the lateral line placode can be distinguished by 18 hpf, and by 48 hpf, the two neuromasts later found to become the otolith have fully developed, and further neuromasts start migration and development (Nuñez et al. 2009; Thomas et al. 2015). Early movement is based on random neuronal firing rather than organized behavior (Kokel et al. 2013), whereas the embryo begins expressing organized coiling responses to head touches by 21 hpf and tail stimuli only elicited a response at 27 hpf (Saint-Amant and Drapeau 1998). This development takes place during the time window of the coiling assay and might explain higher frequencies of movements in group-reared embryos than in separately reared embryos. The movement initiated by one embryo is easily transmitted through, e.g., the aqueous medium or via direct contact, stimulating other embryos, which may then also initiate muscular contractions (Saint-Amant and Drapeau 1998). Other fish species have been found to use the secretion of hatching enzymes or temporal patterns of vibration as cues to initiate clutch-wide hatching (Yamagami 1981; Warkentin et al. 2006), thus further underlining that there are modes of communication between embryos prior to hatching. These findings, together with the presented literature hence support the theory that the lateral line is likely a key factor in the synchronization of hatching and early movement patterns of embryos.

## Conclusions

The present study aimed to improve the understanding of how rearing conditions may impact the effects of developmental neurotoxins in the zebrafish embryo coiling assay. To this end, the known neurotoxicant rotenone was selected to assess how grouped and separately reared zebrafish embryos expressed the impact of exposure over time. The present data showed that rotenone affected the embryonic development of zebrafish in the FET test, and the exposure induced measurable changes in behavioral parameters. This work further highlighted that different rearing conditions under otherwise unchanged experimental conditions led to notable differences in the measured behavior endpoints, leading to the hypothesis that the expression of the behavior may be, in part, linked to external stimuli. While the development of the lateral line has been studied admissibly (Metcalfe et al. 1985; Nuñez et al. 2009; Chitnis et al. 2012; Thomas et al. 2015), gaps remain when it comes to the question of how sensitive and active neuromasts are during the development of the lateral line and whether egg-to-egg transmission of locomotion is possible at that stage. Rotenone has been shown to express neurotoxic effects on zebrafish embryos, yet modifying one parameter in an otherwise well-established neurotoxicity test such as the coiling assay may lead to changes in behavior influenced by the proximity between individual embryos. The findings presented here outline that even when exposed to increased concentrations of rotenone within the EC range of the FET test, differences in the behavioral expression of exposure-induced neurotoxicity were visible between different rearing conditions. As these differences in behavior were also noted in the control groups between the different rearing conditions, our work further underlines the complex dependence of the outcome of behavior assays on experimental (and external) parameters.

## Supplementary Information

Below is the link to the electronic supplementary material.

Supplementary file1 (DOCX 29 KB)

Supplementary file2 (PPTX 206275 KB)

## Data Availability

The data in the publication can be found in the provided supplementary material. Original datasets of the current study and analyses generated are available in the BioStudies repository (https://wwwdev.ebi.ac.uk/biostudies/EU-ToxRisk/).

## Abbreviations

AChE: Acetylcholine (esterase)
ANOVA: Analysis of variance
DCA: 3,4-Dichloroaniline
DMSO: Dimethyl sulfoxide
(D)NT: (Developmental) neurotoxicity
EC: Effect concentration
FET test: Fish embryo acute toxicity test
GABA: Gamma-aminobutyric acid (γ-Aminobutyric acid)
Hpf: Hours post-fertilization
LC: Lethal concentration
OECD TG: Organization for Economic Co-operation and Development test guideline
REACH: Registration, Evaluation, Authorization and Restriction of Chemicals
