# Supplementary material for: Rearing conditions (isolated versus group rearing) affect rotenone-induced changes in the behavior of zebrafish (Danio rerio) embryos in the coiling assay
Source: Environ Sci Pollut Res Int. 2024 Sep 6;31(43):55624–35. doi: 10.1007/s11356-024-34870-x (PMC11415484; doi:10.1007/s11356-024-34870-x)
Supplement: Supplementary file 1 — Supplementary file1 (DOCX 29 KB) [file 11356_2024_34870_MOESM1_ESM.docx]

**Rearing conditions (isolated *versus* group rearing) affect rotenone-induced changes in the behavior of zebrafish (*Danio rerio*) embryos in the coiling assay**

Rebecca von Hellfeld ^a, b,^ *, Christoph Gade ^a, b^, Marcel Leist ^c, d^, Thomas Braunbeck ^b,^ *

^a^ School of Biological Sciences, University of Aberdeen, AB24 3UU Aberdeen, UK

^b^ Current affiliation: Centre for Organismal Studies, Aquatic Ecology and Toxicology, University of Heidelberg, 69120 Heidelberg, Germany

^c^ In Vitro Toxicology and Biomedicine, Department inaugurated by the Doerenkamp-Zbinden Foundation, University of Konstanz, 78457 Konstanz, Germany

^d^ CAAT Europe, University of Konstanz, 78457 Konstanz, Germany

* Corresponding author

**− Supplementary materials –**

**SI 1**. Percentage of zebrafish (*Danio rerio*) embryos analyzed per concentration/time point (n = 3; 20 individuals per concentration and replicate) for group-reared zebrafish embryos.

**SI 2**. Video recording of replicate 3 at 47 hpf of group reared coiling experiment. Data as shown in Figure 2 illustrate the excessive movement leading to the exclusion of data in rotenone exposure experiments. Wells indicated with red rings were completely excluded due to hyperactivity. In some cases, only some individuals were excluded from the analysis, which is indicated by the red ovals/smaller circle. The video has been sped up x4 and is thus only 2 minutes long

**See separate PowerPoint file.**

**SI 3**. *p*-Values for the difference between zebrafish embryos reared separately or in groups and the corresponding negative controls for coiling assay replicates noted as statistically significant in a one-way ANOVA-on-ranks with a Dunn’s *post hoc* test (n = 3, 20 individuals per concentration/replicate). At least 2 replicates had to prove statistically positive at a level of significance of at least *p* ≤ 0.05. In the case of the runs providing identical levels of significance, the *p*-value was listed once.

|  | **Mean burst duration [seconds]** | | | **Mean burst count per minute** | | |
| --- | --- | --- | --- | --- | --- | --- |
|  | **Concentration** | **hpf** | ***p*-Value(s)** | **Concentration** | **hpf** | ***p*-Value(s)** |
| **Grouped rearing** | 20.3 nM | 26 | ≤ 0.01, ≤ 0.0001 | 20.3 nM | 21 | ≤ 0.05, ≤ 0.0001 |
|  |  | 27 | ≤ 0.01 |  | 22 | ≤ 0.0001 |
|  |  |  |  |  | 23 | ≤ 0.01, ≤ 0.0001 |
|  |  |  |  |  | 26 | ≤ 0.01, ≤ 0.0001 |
|  |  |  |  |  | 27 | <0.01 |
|  |  |  |  |  | 46 | ≤ 0.01, ≤ 0.0001 |
|  |  |  |  |  | 47 | ≤ 0.01, ≤ 0.0001 |
| **Separated rearing** | 1.0 nM | 22 | ≤ 0.05. ≤ 0.001 | 1.0 nM | 22 | ≤ 0.01, ≤ 0.0001 |
|  | 10.1 nM | 21 | ≤ 0.05 |  |  |  |

**SI 4**. *p*-Values for the difference between zebrafish embryos reared separately or in groups and the corresponding negative controls for coiling assay replicates between 37 and 38 hpf noted as statistically significant in Wilcoxon match-pairs signed rank test (n = 3, 20 individuals per concentration/replicate). At least 2 replicates had to prove statistically positive at a level of significance of at least *p* ≤ 0.05. In the case of the runs providing identical levels of significance, the *p*-value was listed once.

|  | **Burst count per minute** | | **Mean burst duration [seconds]** | |
| --- | --- | --- | --- | --- |
|  | **Group reared** | **Individually reared** | **Group reared** | **Individually reared** |
| **0.1% DMSO** | ≤ 0.05 |  | ≤ 0.005 |  |
| **1 nM** |  |  | ≤ 0.05 |  |
| **10.1 nM** |  | ≤ 0.05 |  |  |
| **20.3 nM** | ≤ 0.005 | ≤ 0.05 |  |  |
| **Overall** | ≤ 0.0001 | ≤ 0.05 | ≤ 0.00011 |  |

**SI 5.** *p*-Values for the difference between zebrafish embryos reared separately or in groups and the corresponding negative controls for coiling assay replicates between 37 and 28 hpf noted as statistically significant in a Mann-Whitney U-test (n = 3, 20 individuals per concentration/replicate). At least 2 replicates had to prove statistically positive at a level of significance of at least *p* ≤ 0.05. In the case of the runs providing identical levels of significance, the *p*-value was listed once.

| **Mean burst duration** | | **Mean burst count per minute** | |
| --- | --- | --- | --- |
| **Time (hpf)** | ***p*-Value(s)** | **Time (hpf)** | ***p*-Value(s)** |
| 21 | ≤ 0.0001 | 21 | ≤ 0.0001 |
| 22 | ≤ 0.01, ≤ 0.0001 | 22 | ≤ 0.01, ≤ 0.0001 |
| 23 | ≤ 0.05, ≤ 0.0001 | 23 | ≤ 0.0001 |
| 24 | ≤ 0.0001 | 24 | ≤ 0.0001 |
| 25 | ≤ 0.0001 | 25 | ≤ 0.01, ≤ 0.0001 |
| 26 | ≤ 0.0001 | 26 | ≤ 0.05, ≤ 0.0001 |
| 27 | ≤ 0.01, ≤ 0.0001 | 32 | ≤ 0.01, ≤ 0.0001 |
| 28 | ≤ 0.01, ≤ 0.0001 | 33 | ≤ 0.0001 |
| 29 | ≤ 0.0001 | 34 | ≤ 0.05, ≤ 0.0001 |
| 30 | ≤ 0.05, ≤ 0.0001 | 36 | ≤ 0.05, ≤ 0.01 |
| 31 | ≤ 0.01, ≤ 0.0001 | 38 | ≤ 0.05, ≤ 0.0001 |
| 32 | ≤ 0.0001 | 40 | ≤ 0.05, ≤ 0.0001 |
| 33 | ≤ 0.01, ≤ 0.0001 | 41 | ≤ 0.01 |
| 35 | ≤ 0.01, ≤ 0.05 | 42 | ≤ 0.01, ≤ 0.0001 |
| 36 | ≤ 0.05, ≤ 0.0001 | 43 | ≤ 0.0001 |
| 38 | ≤ 0.01, ≤ 0.05 | 44 | ≤ 0.01, ≤ 0.0001 |
| 39 | ≤ 0.01 | 45 | ≤ 0.0001 |
| 40 | ≤ 0.01 | 46 | ≤ 0.0001 |
| 41 | ≤ 0.05, ≤ 0.0001 | 47 | ≤ 0.0001 |
| 42 | ≤ 0.0001 |  |  |
| 43 | ≤ 0.0001 |  |  |
| 44 | ≤ 0.04, ≤ 0.01, ≤ 0.0001 |  |  |
| 45 | ≤ 0.0001 |  |  |
| 46 | ≤ 0.0001 |  |  |
| 47 | ≤ 0.0001 |  |  |
